# Nursing documentation and its relationship with perceived nursing workload: a mixed-methods study among community nurses

**DOI:** 10.1186/s12912-022-00811-7

**Published:** 2022-01-28

**Authors:** Kim De Groot, Anke J. E. De Veer, Anne M. Munster, Anneke L. Francke, Wolter Paans

**Affiliations:** 1 Netherlands Institute for Health Services Research (Nivel), PO Box 1568, 3513CR Utrecht, The Netherlands; 2 Thebe Wijkverpleging [Home care organization], Lage Witsiebaan 2a, 5042DA, Tilburg, The Netherlands; 3 Nursing Science, Programme in Clinical Health Sciences, University Medical Centre Utrecht, PO Box 85500, 3508GA Utrecht, The Netherlands; 4 Department of Public and Occupational Health, Amsterdam Public Health Research Institute, Amsterdam University Medical Centre and Vrije Universiteit Amsterdam, Van der Boechorststraat 7, 1081BT Amsterdam, The Netherlands; 5 Research Group Nursing Diagnostics, School of Nursing, Hanze University of Applied Sciences, Petrus Driessenstraat 3, 9714CA Groningen, The Netherlands; 6 Department of Critical Care, University Medical Centre Groningen, PO Box 30.001, 9700RB Groningen, The Netherlands

**Keywords:** Documentation burden, Electronic health record, Home care, Mixed-methods research, Nursing documentation, Nursing process, Nursing workload, User-friendliness

## Abstract

**Background:**

The time that nurses spent on documentation can be substantial and burdensome. To date it was unknown if documentation activities are related to the workload that nurses perceive. A distinction between clinical documentation and organizational documentation seems relevant. This study aims to gain insight into community nurses’ views on a potential relationship between their clinical and organizational documentation activities and their perceived nursing workload.

**Methods:**

A convergent mixed-methods design was used. A quantitative survey was completed by 195 Dutch community nurses and a further 28 community nurses participated in qualitative focus groups. For the survey an online questionnaire was used. Descriptive statistics, Wilcoxon signed-ranked tests, Spearman’s rank correlations and Wilcoxon rank-sum tests were used to analyse the survey data. Next, four qualitative focus groups were conducted in an iterative process of data collection - data analysis - more data collection, until data saturation was reached. In the qualitative analysis, the six steps of thematic analysis were followed.

**Results:**

The majority of the community nurses perceived a high workload due to documentation activities. Although survey data showed that nurses estimated that they spent twice as much time on clinical documentation as on organizational documentation, the workload they perceived from these two types of documentation was comparable. Focus-group participants found organizational documentation particularly redundant. Furthermore, the survey indicated that a perceived high workload was not related to actual time spent on clinical documentation, while actual time spent on organizational documentation *was* related to the perceived workload. In addition, the survey showed no associations between community nurses’ perceived workload and the user-friendliness of electronic health records. Yet focus-group participants did point towards the impact of limited user-friendliness on their perceived workload. Lastly, there was no association between the perceived workload and whether the nursing process was central in the electronic health records.

**Conclusions:**

Community nurses often perceive a high workload due to clinical and organizational documentation activities. Decreasing the time nurses have to spend specifically on organizational documentation and improving the user-friendliness and intercommunicability of electronic health records appear to be important ways of reducing the workload that community nurses perceive.

## Background

Clinical nursing documentation is essential in letting nurses continuously reflect on their choice of interventions for patients and the effects of their interventions. Therefore, it is vital to the quality and continuity of nursing care [1, 2]. Nursing documentation can be described as a reflection of the entire process of providing direct nursing care to patients [3–5]. Consequently, there is international consensus that clinical nursing documentation has to reflect the phases of the nursing process, namely assessment, diagnosis, care planning, implementation of interventions and evaluation of care or – if relevant – handover of care [2, 3, 6–8].

Despite the evident importance of nursing documentation, time spent on documentation can be substantial and therefore it can be experienced as onerous for nurses. Research indicates documentation time has reached an extreme form [9–11]. Even though the actual time spent by nurses on documentation varies internationally, it is a substantial part of the work of nurses [12, 13]. For example, in Canada nurses spend about 26% of their time on documentation [14], in Great Britain 17% [15] and in the USA percentages vary from 25% to as much as 41% [16, 17]. In the Netherlands, nursing staff reported spending an average of 10.5 hours a week on documentation [18], which means they spend about 40% of their time on documentation.

The variation between countries in nurses’ time spent on documentation may be related to differences in electronic health records and the way in which handovers are organized. However, the variation may also be the result of a lack of clarity about what qualifies as documentation [19, 20]. Some studies used the term ‘documentation’ for activities that were directly related to individual patient care, e.g. drawing up a care plan or writing progress reports [16, 17]. Other studies used ‘documentation’ as an umbrella term that included ‘non-patient-care-related’ documentation as well, such as recording hours worked or recording data for the planning of personnel [18, 20].

A conceptual overview from the Organisation for Economic Cooperation and Development (OECD) provides more conceptual clarity in the various types of documentation [12]. The OECD states that documentation generally can be divided into clinical documentation and documentation regarding organizational and financial issues. Clinical documentation refers to documentation in the electronic health records of individual patients, e.g. about the patient’s medical condition and about the care provided by healthcare professionals. The OECD uses the term ‘organizational documentation’ to refer to the documentation of issues regarding personnel planning and coordinating different shifts, for instance. Documentation such as recording hours worked for the purpose of billing and insurance are categorized by the OECD as financial documentation [12].

There are indications that organizational and financial documentation in particular has increased in the last decade, which might be explained by the rising demand for accountability and efficiency of care [21]. Since documenting organizational and financial issues is not directly related to patient care, these aspects of documentation might be perceived negatively by nurses [22]. In contrast, nurses might be more open to clinical documentation since this documentation is essential to high-quality nursing care [1, 2, 23]. Moreover, according to professional standards and guidelines, clinical documentation should be considered as an integral part of providing nursing care [24–26].

Still, lengthy clinical documentation might be challenging for nurses as well. According to Baumann, Baker [27], Moore, Tolley [28] the implementation of electronic health records for individual patients appeared to increase the observed time that nurses spend on clinical documentation. Yet their findings were inconclusive, since long-term follow-up studies indicated decreasing documentation time once nurses became familiar with the electronic health records [27]. However, other studies indicated that the setup for the electronic health records does not always match nurses’ routines and can therefore be a potential source of perceived time pressure among nurses [29, 30]. Yet when the electronic health records follow the phases of the nursing process, this might be supportive for nurses’ clinical documentation [31].

Nurses’ time pressure and nursing workload have received significant interest, in part because nursing shortages are a problem internationally [32]. Research often focusses only on the objective nursing workload, measured and expressed in actual time spent caring for a patient and/or staffing ratios [33]. However, nurses’ emotional or perceived workload might not always correspond to their objective workload [34]. But the perceived workload of nurses and the related factors is a rather unexplored area. For instance, it was unknown to date if perceived workload is associated with specific types of documentation activities and the actual time spent on these activities.

In line with the above-mentioned conceptual overview from the OECD [12] and from a nursing perspective, it seems relevant to make a distinction between different types of documentation activities. On the one hand, there is clinical documentation, which directly concerns the nursing care for individual patients. On the other hand, there is organizational and financial documentation; this is documentation that is mainly relevant for care organizations, management, policymakers and/or health insurers. In the Dutch context, clinical documentation often includes care needs assessment information, a care plan structured according to the phases of the nursing process, daily evaluation reports concerning the care given, and the handover of care. Organizational and financial documentation often concerns records of hours worked, expense claims for medical aids, reports on incidents with patients and/or employees, internal audits, and measurements of employee satisfaction and/or patient satisfaction.

To date it was unclear whether specific types of documentation are associated with a high perceived nursing workload. Distinguishing between types of documentation may provide more insight into the possible relationship between documentation and perceived nursing workload.

Furthermore, we used a mixed-methods approach to gain a deeper understanding, with a quantitative survey followed by qualitative focus groups. The quantitative data provided a broad and representative picture of the possible presence of a relationship between perceived workload and documentation activities. However, the reasons *why* community nurses felt the specific documentation activities increased their workload became clearer from the qualitative data. Combining the findings from these two methods resulted in a credible and in-depth picture of the relationship between documentation activities and perceived nursing workload. This enabled specific recommendations to be made that can help reduce the workload of nurses.

Such insights are relevant in particular for the home-care setting, since a previous survey showed that community nurses reported spending even more time on documentation compared with nurses working in other settings [18]. In addition, most studies on the documentation burden focus solely on the hospital setting, e.g. the studies of Collins, Couture [35] and Wisner, Lyndon [30].

Therefore, the study presented here aimed to gain insight into community nurses’ views on a potential relationship between clinical and organizational documentation and the perceived nursing workload (in this study, ‘organizational documentation’ includes financial documentation). The research questions guiding the present study were:

(a) Do community nurses perceive a high workload due to clinical and/or organizational documentation? (*survey and focus groups*), (b) If so, is their perceived workload related to the time they spent on clinical and/or organizational documentation? (*survey*).
Is there a relationship between the extent to which community nurses perceive a high workload and (a) the user-friendliness of electronic health records (*survey and focus groups*), and (b) whether the nursing process is central in the electronic health records (*survey and focus groups*)?

## Methods

### Design

A convergent mixed-methods design was used, in which a quantitative survey with qualitative focus groups were combined to develop in-depth understanding of the relationship between documentation activities and perceived nursing workload [36, 37]. This design has been proven to be particularly useful for achieving a deep understanding of relationships [36, 38]. First, the quantitative survey was performed and findings from this quantitative component were subsequently enriched by the findings of the qualitative focus groups [37, 38].

### Participants

#### Survey participants

The nurses who were sent the online survey were participants drawn from a Dutch nationwide research panel known as the Nursing Staff Panel (https://www.nivel.nl/en/panel-verpleging-verzorging/nursing-staff-panel). Members of the Nursing Staff Panel are primarily recruited through a random sample of the population of Dutch healthcare employees provided by two pension funds [4]. In addition, members are recruited through snowball sampling and open calls on social media. All members had given permission to be approached regularly to answer questions about their experiences in nursing practice. For this study, the survey was sent by email to all 508 community nurses who were members of the Nursing Staff Panel. Since this is a nationwide panel, respondents worked in a variety of organizations across the Netherlands. To increase the response rate, two electronic reminders were sent to nurses who had not yet responded.

This paper focusses on community nurses and electronic nursing documentation; therefore only respondent nurses who met the following criteria were included in the analysis: 1) being a registered nurse with a bachelor’s degree or a secondary vocational qualification in nursing; 2) working in home care; 3) using electronic health records. We excluded 24 respondents who did not meet these criteria.

#### Focus-groups participants

Focus-group participants were recruited through the professional network of two authors (KdG and AM), open calls on social media (LinkedIn and Facebook), and through snowball sampling. Nurses were eligible to participate in a focus group if they met the same inclusion criteria as used for the survey participants. Purposive sampling was applied to obtain variation among participants regarding the educational level, age and standardized terminology used in the electronic health records. None of the participants of the focus groups had also participated in the survey.

Since the focus groups were in addition to the survey, we expected a priori that four focus groups would be enough to reach data saturation. This expectation was met, as the last focus group produced no new insights that were relevant for answering the research questions.

### Data collection

#### The survey

The survey data were collected from June to July 2019. We used an online survey questionnaire that mostly consisted of self-developed questions as, to our knowledge, no instrument was available that included questions on both clinical documentation and organizational documentation. The extent to which nurses perceived a high workload was measured using a five-point scale (1 = ‘never’ to 5 = ‘always’). We distinguished between a high workload due to clinical documentation and a high workload due to organizational documentation. We included financial documentation in our definition of organizational documentation. In the questionnaire we explained the content of the two types of documentation. Respondents were then asked to estimate the time they spent on the two types of documentation.

Next, two questions focussed specifically on clinical documentation, namely whether the electronic health record of individual patients was user-friendly and whether the nursing process was central in this record. These questions were derived from the ‘Nursing Process-Clinical Decision Support Systems Standard’, an internationally accepted and valid standard for guiding the further development of electronic health records [31].

The entire questionnaire was pre-tested for comprehensibility, clarity and content validity by nine nursing staff members. Based on their comments, the questionnaire was modified, and a final version produced. A translation of the part of the questionnaire with the 11 questions relevant for this paper can be found at: https://documenten.nivel.nl/translated_questionnaire.pdf.

#### Focus groups

After the survey, we conducted four qualitative focus groups from February to May 2020. Each group consisted of six or eight community nurses, with a total of 28 community nurses. These focus groups were performed in order to deepen and refine the insights gained from the survey data.

The focus groups were led by two authors (KdG and AM) and supported by an interview guide with open questions, see Table 1. The questions were inspired by the results of the survey data, e.g. they addressed how community nurses perceived clinical and organizational documentation in relation to their workload, or how community nurses experienced the user-friendliness of electronic health records.

**Table 1:** Interview guide

1.	Do you experience a relationship between workload and documentation activities? If so, can you explain?
2.	What do you think about the amount of time you spent on documentation activities?
3.	Can you tell us about your experiences with organizational documentation activities related to your perceived workload? By organizational documentation activities we mean documentation that is mostly relevant for care organizations, managers and policymakers, such as records of hours worked, expense claims, or reports on incidents.
4.	Can you tell us about your experiences with clinical documentation activities related to your perceived workload? By clinical documentation activities we mean documentation which directly concerns the nursing care for individual patients, such as drawing up a care plan, documenting daily evaluation reports or the handover of care.
5.	How do you experience documentation in the electronic health record?
6.	How do you experience the user-friendliness of the electronic health record that you work with?
7.	How do you experience the use of various electronic systems for your documentation activities? By electronic systems you can think of electronic health records, systems for records of hours worked, and/or systems for expense claims.
8.	How do you experience the documented handover of care to other healthcare professionals, such as general practitioners and hospital nurses? Can you think of improvements regarding these documentation activities?

Initially, we aimed to conduct all the focus groups face-to-face at the care organizations’ offices. However, after one face-to-face focus group we had to switch to online focus groups due to the COVID-19 pandemic. Online focus groups in which participants post written responses in a secure online discussion site have been proven to be an appropriate alternative for face-to-face focus groups [39–41]. In fact, the online focus groups had several advantages, such as providing participants with the ability to access, read and respond to posts at a place and time most convenient to them [40, 41]. This was particularly advantageous for nurses during the pandemic.

Each online focus group was conducted within a set period of 2 weeks. Two authors (KdG and AM) acted as moderators by regularly checking the responses and posting new questions every 2 days, except in the weekend. The analysis of the transcripts has shown that the findings from the online focus groups were comparable to those from the face-to-face focus group.

### Data analysis

#### Analysis of the survey

Descriptive statistics were used to describe the background characteristics of the respondents and to answer the first and second research questions. Wilcoxon signed-ranked tests were conducted to answer the first research question (1a), since the two variables measuring the perceived workload were ordinal and the two variables measuring the estimated time spent on documentation were not normally distributed. Next, the potential relationships between perceived workload and time spent on documentation (research question 1b) were examined using Spearman’s rank correlations. Wilcoxon rank-sum tests were conducted to examine associations between perceived workload and user-friendliness (research question 2a) and the nursing process (research question 2b). The level for determining statistical significance was 0.05. Analyses were conducted using STATA, version 16.1.

#### Analysis of the focus groups

The audio recording of the face-to-face focus group was transcribed verbatim. Transcripts from the online focus groups were taken directly from the discussion site.

The focus-group transcripts were analysed using an iterative process of data collection - data analysis - more data collection. Within this process, the six steps of thematic analysis were followed, namely becoming familiar with the data, generating initial codes, searching for themes, reviewing themes, defining and naming themes, and reporting [42].

The transcripts of all the focus groups were analysed by two authors (KdG and AM). They refined their analyses in discussions together and with two other authors (AF and WP), which ultimate led to consensus about the main themes. This triangulation of researchers was used to increase the quality and trustworthiness of the analysis [43]. Moreover, ‘peer debriefing’ was applied with a group of peer researchers who were not involved in the study. In addition, confirmability of the findings was enhanced by including verbatim statements made by participants in the results section of this paper. Furthermore, the quality of the reporting was ensured by following the guidelines in ‘Good Reporting of A Mixed Methods Study’ [44].

#### Data integration

By integrating data from the quantitative and qualitative components, an in-depth and credible picture was obtained of the relationship between specific documentation activities and perceived nursing workload [36, 37]. The data were integrated using two integration approaches. Firstly, we compared the data from the survey and focus groups in the analysis process, in discussions among the authors, and in the ‘Discussion’ section of this article. This is referred to as the ‘merging’ approach [37]. For instance, the survey result on how many nurses perceived a high workload from clinical documentation activities was compared to the focus groups results on nurses’ views as to why they did or did not perceive a high workload from these activities. Secondly, integration through narratives was performed when reporting the results. Hereby we used a ‘weaving’ approach in which we brought the findings from the quantitative survey and qualitative focus groups together on a thematic basis and arranged them according to the research questions [37].

### Ethical considerations

The study was conducted in compliance with the principles of the General Data Protection Regulation, by strictly safeguarding the anonymity of the participants. Formal approval from an ethics committee was not required under the applicable Dutch legislation on medical scientific research as participants were not subjected to procedures and were not required to follow rules of behaviour (see https://english.ccmo.nl/investigators/legal-framework-for-medical-scientific-research/your-research-is-it-subject-to-the-wmo-or-not).

Participants in the survey had all consented to being sent and completing surveys on a regular basis on topics directly related to their work when they signed up as members of the Nursing Staff Panel. Potential participants of the focus groups were informed about the study in an information letter. If desired, they could obtain additional verbal information. All participants signed an informed consent form before the focus groups started.

All methods were applied in accordance with relevant guidelines and regulations.

## Results

### Participants

A total of 195 community nurses completed the questionnaire (response rate 38.4%). Since a substantial group did not respond, we conducted non-response analyses. We found no statistically significant differences between the respondents and non-respondents regarding gender, level of education and number of hours employed. We did however see a difference in age: the respondents were somewhat older (mean age 49.8 years) than the non-respondents (mean age 44.3 years). We reflect on the relatively high age of the survey respondents in ‘Limitations and strengths’ section.

A total of 28 community nurses participated in the four focus groups. The characteristics of the participants are presented in Tables 2 and 3.

**Table 2:** Survey participants’ characteristics

Characteristics	Survey participants (*n* = 195)
**Age [mean (SD)]**	50.1 (11.5)
**Gender [n (%)]**	
Female	182 (93.3%)
Male	13 (6.7%)
**Level of education [n (%)]**	
Registered nurse secondary vocational qualification	86 (44.1%)
Registered nurse bachelor’s degree	109 (55.9%)
**Standardized terminology [n (%)]**^**#**^	
Omaha System	164 (84.1%)
NANDA-I NIC NOC	21 (10.8%)
Other standardized nursing terminologies	13 (6.7%)
No standardized terminology	6 (3.1%)
I don’t know	7 (3.6%)
**Number of hours employed [mean (SD)]**	25.3 (7.1)

^#^Multiple answers possible

**Table 3:** Focus-groups participants’ characteristics

Characteristics	Focus group participants (*n* = 28)
**Age [mean (SD)]**	33.7 (11.3)
**Gender [n (%)]**	
Female	26 (92.8%)
Male	2 (7.2%)
**Level of education [n (%)]**	
Registered nurse secondary vocational qualification	6 (21.4%)
Registered nurse bachelor’s degree	22 (78.6%)
**Standardized terminology [n (%)]**	
Omaha System	27 (96.4%)
NANDA-I NIC NOC	1 (3.6%)

### Perceived workload due to documentation and time spent documenting

More than half of the community nurses in the survey said that they perceived a high workload due to clinical and/or organizational documentation, see Table 4. A majority (52.4%) said that they regularly to always experienced a high workload due to clinical documentation. Regarding organizational documentation, 58% of the nurses reported a high perceived workload. No statistically significant differences in perceived workload were found between the two types of documentation (Wilcoxon signed-ranked test: *p* = 0.124). In other words, nurses were just as likely to experience a high workload due to clinical documentation as due to organizational documentation.

**Table 4:** Community nurses’ perceived workload due to documentation and estimated time spent on documentation

Variables	Clinical documentation (*n* = 195)	Organizational documentation (*n =* 195)	*p* value
**Perceived high workload [n (%)]**			0.124
Never	8 (4.1%)	10 (5.1%)	
Rarely	85 (43.6%)	72 (36.9%)	
Regularly	68 (34.9%)	75 (38.5%)	
Often	27 (13.9%)	31 (15.9%)	
Always	7 (3.6%)	7 (3.6%)	
**Estimated time per week spent on documentation [Mean (SD; median)]**	8.0 (6.0; 6.0)	3.6 (4.0; 2.0)	< 0.000

Community nurses in the survey estimated that they spent on average 8.0 (SD 6.0; median 6.0) hours a week on clinical documentation. They estimated that they spent significantly less time on organizational documentation, namely on average 3.6 (SD 4.0; median 2.0) hours a week (Wilcoxon signed-ranked test: *p* < 0.000).

Looking at clinical documentation, no statistically significant correlation was found between nurses’ estimated time spent on this type of documentation and their perceived high workload (Spearman’s rank correlation 0.135; *p* = 0.058). However, looking at organizational documentation, a statistically significant moderate correlation was found between time spent on documentation and perceived high workload (Spearman’s rank correlation 0.375; *p* < 0.000). This showed that nurses who spent more time on organizational documentation were more likely to perceive a high workload.

In general, the community nurses participating in the qualitative focus groups experienced a high workload due to documentation as well. They described organizational documentation in particular as cumbersome, redundant and too repetitive in nature. Even though nurses believed that a high workload in general is common among community nurses, they did see documentation as one of the causes for their high workload.

*“You are already busy sorting out all the shifts, all the patients who are starting and stopping home care etc. There’s already a high workload. And on top of all that, there are the documentation activities. In our organization, they also want everyone to do refresher courses to keep their registration as a nurse, so you need to register that too. That is another extra documentation burden, and that takes up extra time too.” (Focus group 1, face-to-face).*

A general picture that emerged from the focus groups is that organizational documentation was a key reason for community nurses’ perceived workload, while this was less so for clinical documentation. Community nurses in the focus groups said that they often failed to see the added value of organizational documentation for their patients and themselves. Therefore they had a feeling of frustration with the organizational documentation, associated with a high perceived workload.

*“I think the frustration comes much more from the organizational side. From powerlessness because of all the pointless things you don’t really have time for.” (Focus group 1, face-to-face).*

Focus-group participants mentioned that various rules and regulations imposed by their employers and/or national organizations, such as health insurers, also affected the amount of organizational documentation. They perceived a high workload when they had to register information only for the sake of these rules and regulations.

*“Whenever someone in the organization starts talking about reducing the documentation burden, my blood pressure starts to rise. Then I know for certain that it’ll come back in spades some other way: someone else’s documentation burden will be reduced, but not mine.” (Focus group 1, face-to-face).*

Community nurses in the focus groups were more positive about their clinical documentation activities. They found clinical documentation necessary and useful for providing good nursing care. For them it was evident that this documentation was an important part of their work. Because they saw clinical documentation as directly connected to individual patient care, they were less negative about the time they had to spend on clinical documentation compared with organizational documentation. Some nurses did however mention that documenting the formal care needs assessment (which is a requirement for home care financed by health insurers in the Netherlands) consumed a lot of their time. Still, nurses did not find this kind of documentation burdensome due to the perceived relevance and usefulness of the documentation of the care needs assessment. This was also the case for clinical documentation relating to individual patient care in general.

*“The documentation activities I carry out for my patients are appropriate for my job and the documentation is not an additional burden. On the contrary, that documentation helps me and my fellow nurses to give our patients good, appropriate care.” (Focus group 4, online).*

### Perceived workload and features of electronic health records

Elaborating further on clinical documentation specifically, we explored the perceived workload in relation to two features of the electronic health records, namely user-friendliness and whether the record matches with the nursing process.

#### User-friendliness of electronic health records in relation to workload

Most of the community nurses in the survey agreed that the electronic health records in which they documented information about the nursing care for individual patients were user-friendly (78.8%). A smaller group disagreed (17.6%) and a few did not know (3.6%). The survey participants who answered ‘don’t know’ were excluded from the analysis of the association between user-friendliness and the perceived workload. No statistically significant association was found between how often the nurses perceived a high workload and the user-friendliness of electronic health records (Wilcoxon rank-sum test: *p* = 0.166), see Table 5.

**Table 5:** Association between perceived workload and the user-friendliness of electronic health records

Perceived high workload	The electronic health record I work with is user-friendly		
	Agree (*n* = 152)	Disagree (*n* = 34)	*p* value
Never	6 (4.0%)	2 (5.9%)	0.166
Rarely	72 (47.4%)	12 (35.3%)	
Regularly	51 (33.6%)	11 (32.4%)	
Often	21 (13.8%)	5 (14.7%)	
Always	2 (1.3%)	4 (12.8%)	

As for the user-friendliness of electronic health records the opinions and experiences of the community nurses in the qualitative focus groups were divided. While several community nurses were positive about the user-friendliness of the electronic health records, others were less positive. The latter group said that the limited user-friendliness was one reason why they spent so much time on documentation and experienced a high workload. Elaborating on the limited user-friendliness, nurses in the focus groups explained that some mandatory sections or headings in the electronic health records, e.g. about wound care, cost them too much time. They did not always see the added value of filling in those sections, making this a burdensome activity. Furthermore, nurses stated that the fact that they often had to switch between different sections of the electronic health record was time-consuming and burdensome for them as well.

*“I also find it a pain that you need to search in different sections for a lot of things. The care plan describes that you have to perform wound care according to the wound policy, but the wound policy itself is under a different heading than the care plan. Then the reports about the wound are under the care plan again. And if the patient also needs help with ADL, you have to go back via the care plan again. It all costs extra time and you have to do a lot of clicking.” (Focus group 3, online).*

Focus-group participants also addressed another issue regarding the limited user-friendliness of the electronic health records in relation to their workload. This is the large diversity in electronic systems used within and across care organizations and professionals. For instance, nurses said that they used different systems for documenting wound care and for documenting the medication check. Furthermore, other healthcare professionals, such as general practitioners or pharmacists, often use different electronic systems for their clinical documentation. Community nurses stated that these systems are often not linked to one another, resulting in duplicate documentation activities for nurses and increasing their workload.

*“We have at least a dozen systems and only a few are linked to each other. [...] The systems for communicating with other disciplines and medication systems aren’t linked to one another. Despite the positive discussions, you’re still dependent on the preferences of the pharmacist or GP as to what systems are used. That can lead to you having three different medication systems in one team, for example.” (Focus group 4, online).*

#### Nursing process in electronic health records in relation to workload

In the survey, the majority of community nurses agreed that the nursing process was central in their electronic health records (78.7%). Some nurses disagreed (17.2%) and a few did not know (4.2%). To examine a possible association with workload, survey participants who answered ‘don’t know’ were excluded from this analysis. No statistically significant association was found between a perceived high workload and whether the nursing process was central in the records (Wilcoxon rank-sum test: *p* = 0.542), see Table 6.

**Table 6:** Association between perceived workload and whether the nursing process is central in electronic health records

Perceived high workload	The nursing process is central in the electronic health record		
	Agree (*n* = 151)	Disagree (*n* = 33)	*p* value
Never	6 (4.0%)	2 (6.1%)	0.542
Rarely	63 (41.7%)	16 (48.5%)	
Regularly	55 (36.4%)	8 (24.2%)	
Often	21 (13.9%)	6 (18.2%)	
Always	6 (4.0%)	1 (3.0%)	

Like the survey respondents, virtually all community nurses in the focus groups were positive about how the nursing process was integrated in the electronic health records they worked with.

*“I think we have a very nice system that functions well. [...] I also get sufficient support from this system in my task as a community nurse monitoring the nursing process.” (Focus group 4, online).*

Hence, this feature of the electronic health records was not associated with the workload of the community nurses.

## Discussion

The present study revealed that the majority of community nurses participating in the survey and focus groups perceived documentation as a cause of their high workload. These findings are in line with previous research that indicated that documentation can be burdensome to nurses [9, 10]. Although community nurses spent twice as much time on clinical documentation compared to organizational documentation, the survey showed that community nurses were just as likely to perceive a high workload due to clinical documentation as to organizational documentation. In the focus groups, nurses indicated that organizational documentation in particular was a cause of their high workload. They were more positive about clinical documentation since they experienced that as a meaningful and integral part of the care for individual patients. This view is in line with professional guidelines that describe clinical nursing documentation as an integral part of nursing care for individuals [24–26].

Nevertheless, the survey in particular showed that community nurses often did perceive a high workload due to clinical documentation as well. In the focus groups participants had more opportunity to reflect on and to discuss the value of clinical documentation versus organizational documentation, and this may have resulted in more positive views on clinical documentation.

Still, it is rather surprising that particularly in the survey clinical documentation was associated with a high workload by so many community nurses. Previous research by Fraczkowski, Matson [45];Michel, Waelli [20];Moy, Schwartz [46];Vishwanath, Singh [47];Wisner, Lyndon [30] indicated that electronic clinical documentation is associated with documentation burden by health care professionals. It seems important that all nurses are made aware that clinical nursing documentation is important for providing good patient care. This awareness might reduce nurses’ perceived workload associated with documentation activities. On top of that, further integrating clinical documentation in individual patient care and improvements in the electronic health records are needed [45, 48].

For optimal integration of clinical documentation in patient care, it is important that the electronic health records reflect the phases of the nursing process [6, 31]. However, our study showed no association between the extent of nurses’ perceived workload and whether the electronic health records was following the nursing process. A possible explanation is that most community nurses (78.7%) already found that the nursing process was central in their electronic health records.

A key recommendation for care organizations and software developers is to improve electronic health records in terms of their user-friendliness [4, 31]. Other recent studies also linked the limited usability or user-friendliness of electronic health records to nurses’ perceived time pressure [29, 49]. The community nurses participating in the focus groups also recommended improvements in the user-friendliness of electronic health records and stated that that would reduce their workload. Examples would be removing mandatory sections in electronic health records and working on better communication between systems within and across care organizations and healthcare professionals.

Furthermore, focus-group participants recommended linking the content of the different electronic systems for clinical and organizational documentation so that relevant information only has to be documented once. Other research also indicated that duplication in documentation is a problem for nurses and is accompanied with negative views on documentation [11]. Moreover, studies showed a poor match between different electronic health records both in the digital formats that are used and in the professional vocabulary and standard terminologies used [50, 51]. Improvements in electronic health records, linkages between different electronic systems and more uniformity in language could facilitate information sharing with other healthcare professionals and interdisciplinary care [48, 52].

Another finding in our study was that although clinical documentation was also associated with a high workload, time spent on organizational documentation was considered even more problematic. Unlike clinical documentation, organizational documentation was often seen as pointless. Spending a great deal of time on organizational documentation gave feelings of frustration and a high perceived workload. Our study did not differentiate between different kinds of organizational documentation in terms of the aims of the documentation, e.g. financial accountability for insurers, quality indicators for the Health Inspectorate, safety and quality management for the nurse’s own care organization, etcetera. The association between the specific aims of organizational documentation and nurses’ perceived workload could be a subject for future research. In addition, further research should focus on the integration of clinical documentation in patient care and the user-friendliness of electronic health records.

### Limitations and strengths

A limitation of this mixed-methods study is that the survey participants and focus-group participants differed in age: the focus-group participants were on average younger than the survey participants. We looked at the survey data for a possible correlation between age and perceived workload but did not find statistically significant differences.

A second limitation is that we used a self-developed survey questionnaire. However, we based the questionnaire on relevant literature, including the ‘Nursing Process-Clinical Decision Support Systems Standard’ [12, 31]. Furthermore, we tested the questionnaire in a pilot study for comprehensibility among nursing staff. Hence, we consider the questionnaire to be a comprehensive and content valid instrument to assess nurses’ experiences with documentation in relation to their perceived workload.

A strength of this study was the use of mixed-methods research, which provided a deeper understanding of community nurses’ documentation activities in relation with their perceived workload. The focus groups that were organized after the survey gave additional and more in-depth insights, particularly regarding nurses’ views on the two types of documentation and the user-friendliness of electronic health records.

## Conclusions

The majority of community nurses regularly perceived a high workload due to documentation activities. Although nurses spent twice as much time on clinical documentation as on organizational documentation, the workload they perceived from these types of documentation was comparable. The extent to which nurses perceived a high workload was related to time spent on organizational documentation in particular. Nurses believed spending substantial time on clinical documentation was worthwhile, while spending a great deal of time on organizational documentation led to frustration. Therefore, a reduction in the time needed specifically for organizational documentation is important.

Particularly in the focus groups, nurses highlighted the importance of user-friendly electronic health records in relation to perceived workload. Improving the user-friendliness of electronic health records, improving the intercommunicability of different electronic systems, and further integrating clinical documentation in individual patient care are also recommended as measures to reduce the workload that community nurses perceive from documentation activities.

## Data Availability

The data that support the findings of this study are available from the corresponding author upon reasonable request.
